# Interfacial Interlocking of Carbon Fiber-Reinforced Polymer Composites: A Short Review

**DOI:** 10.3390/polym17030267

**Published:** 2025-01-21

**Authors:** Jong-Hyun Joo, Seong-Hwang Kim, Yoon-Ji Yim, Jin-Seok Bae, Min-Kang Seo

**Affiliations:** 1 Korea Institute of Convergence Textile, Iksan 54588, Republic of Korea; jhj@kictex.re.kr (J.-H.J.); seonghwang@kictex.re.kr (S.-H.K.); 2 Department of Textile System Engineering, Kyungpook National University, Daegu 41566, Republic of Korea; 3 Busan Textile Materials Research Center, Korea Dyeing and Finishing Technology Institute, Busan 46744, Republic of Korea; yjyim@dyetec.or.kr

**Keywords:** surface modification, polymer matrix modification, carbon fibers, interfacial properties

## Abstract

The mechanical properties of the carbon fiber-reinforced polymer composites (CFRPs) are dependent on the interfacial interaction and adhesion between carbon fibers (CFs) and polymer matrices. Therefore, it is crucial to understand how modifying the CFs can influence the properties of these composites. This review outlines recent research progress with a focus on the relationship between the interfacial and mechanical properties of CFRPs and provides a systematic summary of state-of-the-art surface modification techniques. These techniques are divided into four categories: (i) wet, (ii) electrochemical, (iii) dry, and (iv) polymer matrix modifications. Several strategies for enhancing the interfacial interactions and adhesion of CFRPs are discussed, providing insights for future trends.

## 1. Introduction

**Figure 1:**
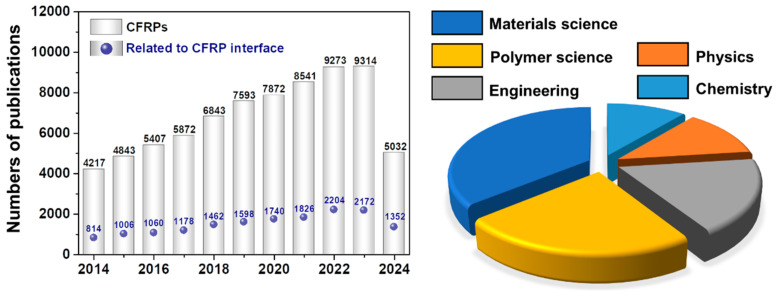
Statistical survey of the literature based on keywords in the field of CFRPs over a 12-year period (2014–2024). The number of publications related to CFRP interfaces is represented by blue dots. Checked on Web of Science as of June 2024.

Carbon fiber-reinforced polymer composites (CFRPs) have broad application potential in the automobile, maritime, and aerospace industries, including the fabrication of sensors and electronic devices, owing to their high specificity, stiffness, low weight, and non-corrosive nature [1,2,3,4,5]. The rapid development of the CFRP industry significantly drives production, consumption, and economic growth while enhancing the overall quality of life [6,7,8]. For instance, fiscal and tax incentives are given to automobile companies to lower CO_2_ emissions from vehicles by reducing their weight and making them more fuel efficient. CFRPs have demonstrated significant possibilities in the light-weighting of automobile systems [9,10,11]. Nevertheless, despite 50 years of research into CFRPs, there is still a large gap between their actual and theoretical mechanical performances. The practical optimization of the CF–matrix interface is the key to solving this critical problem [12,13,14,15,16,17]. Hence, recent research has focused on enhancing the mechanical performance of CFRPs by investigating, understanding, and optimizing the interfacial adhesions between fibers and the matrix. The resultant increasing interest in CFRPs is clearly reflected by the increasing number of publications on this topic over the past decade, as shown in Figure 1.

**Figure 2:**
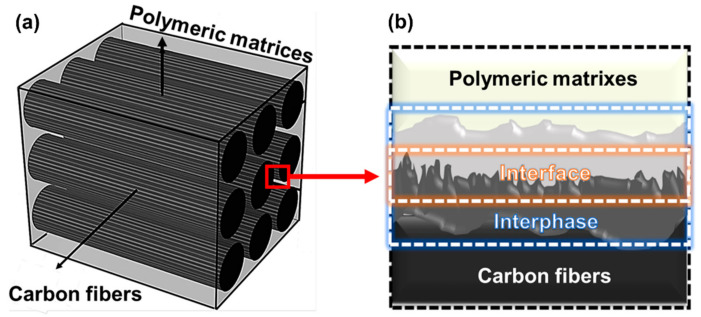
Schematic 3D cross-sections of CFRP interfaces include (**a**) a cross-section and (**b**) the interface and interphases.

According to Drzal’s theory [18], the interfaces within a CFRP are the 2D region between CFs and the polymer matrix. The interphase is the entire 3D region between the two phases (i.e., the polymer matrix and CFs), as shown in Figure 2. The latter includes the 2D interface and wider 3D area extending away from the individual CFs into the polymer matrix [19,20,21,22].

In the CFRPs, the CFs should be firmly embedded within the polymer matrix via interfacial adhesion. This interfacial adhesion is enhanced when the polymeric matrix penetrates surface defects such as valleys, micropits, crevices, or other irregularities in the CFs, as shown by the background in Figure 2b [23,24,25,26]. In addition, the surface properties can be modified by introducing surface functional groups to the CFs [27,28,29,30]. These surface functional groups act as reactive sites and play a crucial role in enhancing the interfacial adhesions between the CF and the polymer matrix [31,32,33,34,35,36,37,38,39]. In addition, a multiscale hierarchy with nano- or micro-scale fillers is an innovative and efficient approach for considerably enhancing the interfacial properties of the CFRP by increasing the interfacial interactions and adhesion between the CFs and the polymer matrix [40,41,42,43]. In most cases, interfacial interaction and adhesion enhance the interfacial properties of the CFRPs [44,45,46].

This review provides a comprehensive discussion of the recent research progress in the surface and structural modification of CFRPs in terms of their interfacial properties. Various surface treatments were classified into wet, electrochemical, and dry treatments. Wet treatments include acidic, amine, silane, and coatings. Electrochemical treatments include chemical vapor deposition (CVD) and electrophoretic deposition (EPD), and dry treatments include plasma and ozone treatments. The modification of the polymer matrix using multiscale methods is additional promising strategy for enhancing the mechanical properties by synergistically integrating nanoscale fillers into the CFRPs. Finally, a comprehensive review of recent advancements in CFRP interfaces is presented, with a special focus on the evolving understanding of the constitutive relationship between interfacial properties and mechanical performance.

## 2. Surface Modifications

### 2.1. Wet Treatments

#### 2.1.1. Acid Treatment

**Figure 3:**
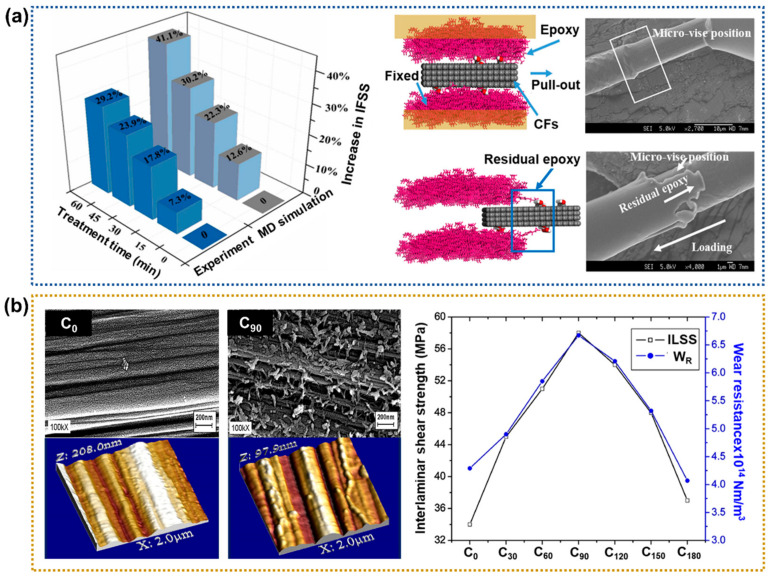
Interfacial properties of acid-treated CFRPs: (**a**) Comparison of the simulated and experimentally measured shear strength (**left**) and the proposed shear mechanism after treatment with a HNO_3_/H_2_SO_4_ solution (**right**); reprinted from Ref. [51], Copyright 2019, Elsevier. (**b**) Surface SEM and AFM images of the untreated CFs (C_0_) and the CFs after treatment with HNO_3_ for 90 min (C_90_) (**left**), and a chart showing the correlation between wear resistance and ILSS according to treatment time (**right**); reprinted from Ref. [52], Copyright 2012, Elsevier.

Acid treatments can modify the surface chemistry and structural characteristics of CFs, leading to enhanced interfacial properties. Concentrated acid imparts oxygen functional groups to the CF surface while improving the surface roughness by inducing expanded voids, crevasses, and pits on the fiber surface [47,48,49,50]. Wang et al. [51] examined the effects of treatment with 3:1 (*v*/*v*) mixtures of concentrated HNO_3_ and H_2_SO_4_ for various treatment times on the interfacial shear energy and shear strength of the epoxy-based CFRPs. In the model simulation, the interfacial shear energy and shear strength increased by 41.1% and 29.9%, respectively, after a 60 min acid treatment. Mechanistically, a molecular dynamics (MD) simulation showed that the oxygen-containing ends of the polymer chains preferentially accumulate on the CF surface. The entire molecular chain is driven towards the CF surface by an interaction with the functional groups thereon, as shown in Figure 3a. The adsorption driving force is mainly the van der Waals force and chemical bond formation. Moreover, more functional groups on carbon fibers enhanced the micro-mechanical interlocking effect at the interface. They suggested that the interfacial properties of the CFRP improve as the acid treatment time increases. Moreover, the oxygen-containing functional groups on the CFs enhance the micro-mechanical interlocking effect at the interface. Tiwari et al. [52] optimized the interfacial interactions between CFs and a polyetherimide (PEI) matrix. They investigated the effects of the HNO_3_-treated CFs on the inter-laminar shear strength (ILSS) and wear resistance of the CFRPs. The oxidation treatment with concentrated HNO_3_ (65–68 wt.%) was used on the CFs for various times (0–180 min) to explore the treatment effect on the fiber–matrix interface. The surface roughness of the CFs increased with extended surface treatment duration, thereby generating additional active sites on the fiber surface, which facilitated improved interfacial adhesion (Figure 3b). Their results suggested that the treatment time of 90 min led to ~74.6% and ~55.2% increases in the ILSS and wear resistance, respectively, compared to the neat CFRPs.

Although these acid treatments are efficient, they continue to have certain drawbacks when applied to CFRPs. For example, the acid treatment can cause defects or reduce the strength of the CFs by generating cracks, flaws, and perforations in the CF surfaces. Hence, future research studies on the acidic modification of CFs should focus on avoiding defects and strength reduction and promoting reinforcement mechanisms.

#### 2.1.2. Amine Treatment

**Figure 4:**
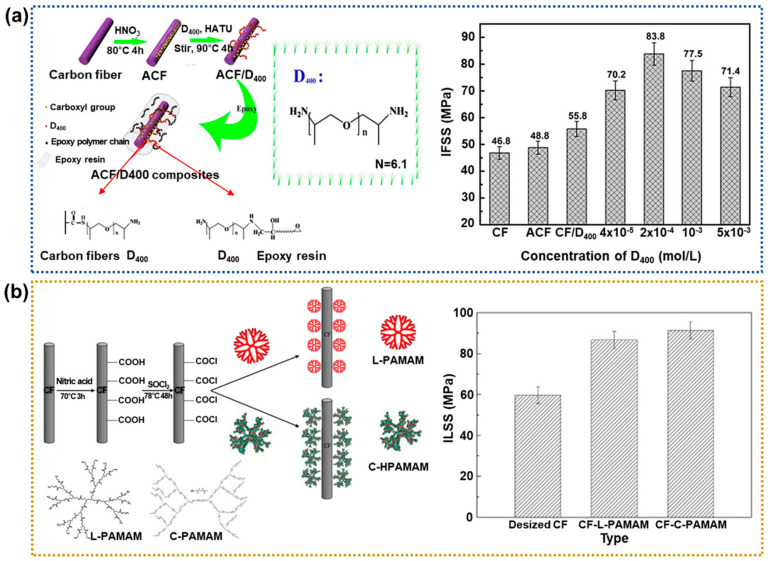
Interfacial properties of amine-treated CFRPs: (**a**) Schematic illustration of poly(oxypropylene) diamines grafted to the CF surface in water (**left**) and a bar chart showing the IFSS values of the CFRPs according to the D400 concentration (**right**); reprinted from Ref. [57], Copyright 2017, Elsevier. (**b**) A schematic diagram (**left**) showing the functionalization progress and molecular structures of two types of CFs, and a bar chart (**right**) showing the ILSS values of the corresponding CFRPs; reprinted from Ref. [58], Copyright 2016, Elsevier.

Researchers have used various amine polymers to modify CFs to enhance the interfacial adhesion of CFRPs [53,54,55,56]. Specifically, the addition of an amine phase as the coupling agent facilitates a reaction with the carboxylic groups on acid-treated CFs to improve the interfacial properties. In addition, amines are used as curing and toughening agents for the polymer matrix because the amino group can provide a chemical bridge between the CFs and the polymer matrix. For example, Wang et al. [57] examined the effects of treatment with various molar concentrations of poly(oxypropylene)diamine (D400) on the interfacial shear strength (IFSS) of the CFRPs. Figure 4a shows a schematic diagram of the chemical grafting of the CFs as a function of the D400 concentration. As a result, the IFSS values of the D400-based CFRPs were increased by up to ~79.1% compared to the neat CFRPs. The authors concluded that D400 can efficiently improve the interfacial interaction of the CFRPs by enhancing the polymer matrix wettability and increasing the interfacial interlocking. Gao et al. [58] examined the effects of poly(amidoamine) (PAMAM)-treated CFs on the ILSS and impact strength of epoxy-based CFRPs. First, PAMAM containing a terminal linear amine (denoted as L-PAMAM) was prepared by repeated reactions between ethanediamine and methyl acrylate. Similarly, PAMAM samples with cyclic terminal groups (denoted as C-PAMAM) were prepared by a reaction of aminoethylpiperazine and methylene-bis-acrylamide. The experimental results suggested that the IFSS and impact strength of the C-PAMAM-treated CFRPs were increased by ~53.1% and 55.8%, respectively, compared to the neat CFRPs (Figure 4b). These results revealed the effectiveness of enhancing the mechanical properties of the CFRPs by controlling the interface between the CFs and epoxy matrix.

In particular, these studies also reported that amine functionalization after an acid treatment can improve the of the CFRPs compared to the acid treatment alone. This is because the acid treatment removes impurities and produces a wide range of oxygen functional groups, including hydroxyl (O–H) and carboxyl (COOH) groups, onto the CF surfaces. Hence, the subsequent amine treatment promotes the cross-linking reaction between the CFs and polymeric matrix by forming amino functional groups, enhancing the interfacial properties. However, the treatment process typically requires a long reaction time, harmful acid–base solutions, and tedious steps.

#### 2.1.3. Silane Coupling Treatment

**Figure 5:**
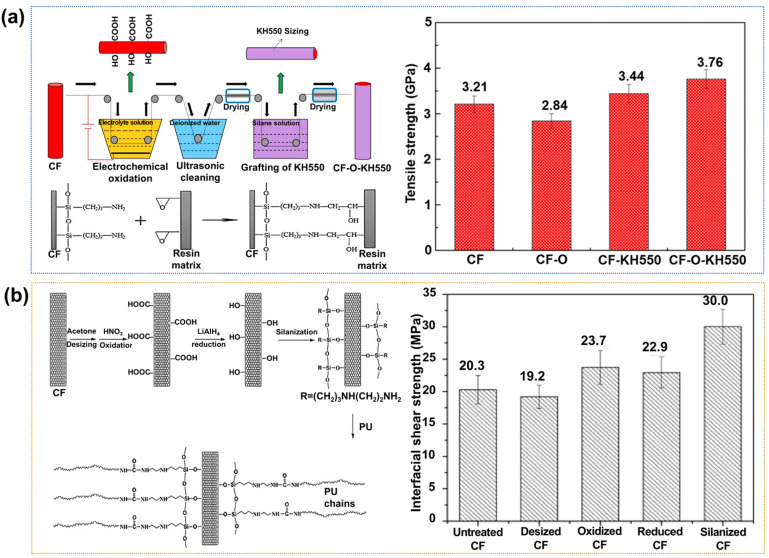
Interfacial properties of silane-treated CFRPs: (**a**) Schematic illustration (**left**) of a two-step silane treatment, and a bar chart (**right**) showing the tensile strengths of the resulting CFRPs; reprinted from Ref. [62], Copyright 2019, Elsevier. (**b**) A schematic diagram (**left**) showing a possible reaction mechanism between CFs and the PU matrix, and a bar chart (**right**) showing the IFSS of CFRP values of various samples; reprinted from Ref. [63], Copyright 2015, Elsevier.

Silane coupling agents have two or more different reactive groups in their molecules [59,60,61]. Of these groups, one type bonds chemically with the CFs, while the other type bonds react chemically with the matrix polymer chains. Wen et al. [62] examined the effects of the electrochemical treatment of the CFs, followed by the grafting of a silane coupling agent (KH550), on the IFSS and tensile strength of the resulting epoxy-based CFRPs (Figure 5a). The KH550 agent could react with oxygen-containing functional groups to generate a cross-linked network. The IFSS and tensile strength values of the KH550-treated CFRPs were increased by up to ~73.1% and 32.3%, respectively, compared to the neat CFRPs. Their results suggested that the introduction of the KH550 silane onto the CF surface resulted in enhanced interfacial interactions, which is advantageous to the penetration of the CFs within the epoxy matrix. Similarly, Jiang et al. [63] examined the effects of a CF surface treatment with [3-(2-aminoethyl)aminopropyl]trimethoxysilane on the ILSS and tensile strength of polyurethane (PU)-based CFRPs. A four-step modification process is performed, including desizing, oxidation, reduction, and silane treatment, as shown in Figure 5b. The experimental results showed that the tensile strength and IFSS values of the silanized CFRPs increased by ~19.4% and 47.9%, respectively, compared to those of the neat CFRPs. The authors reported the mechanical enhancement of the CFRPs to the strong interfacial interactions between the silanized CFs and the PU matrix.

A silane treatment is a promising and versatile strategy because varied silane monomers can be grafted onto the CFs to tailor the interfacial interactions according to the chemical properties. These silane coupling agents can enhance the surface wettability of the CFs, enhancing the compatibility between the CFs and the polymer matrix. On the other hand, the direct introduction of silane coupling agents onto untreated CFs is ineffective because of the poor wettability of the untreated CFs.

#### 2.1.4. Sizing Treatment

**Figure 6:**
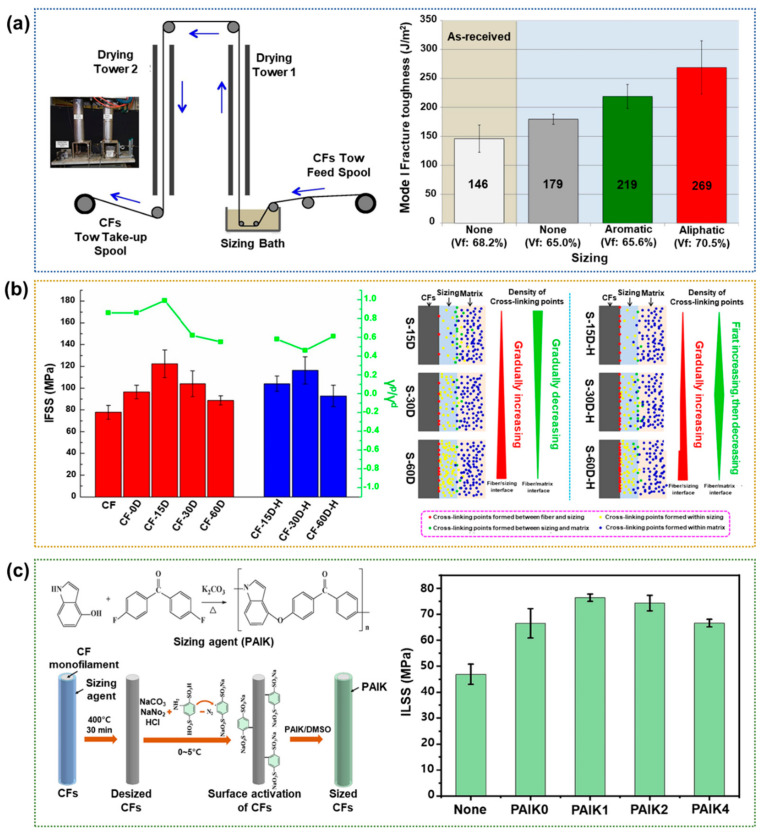
Interfacial properties of CFRPs when treated with sizing agents: (**a**) A photographic image and schematic diagram of a continuous fiber sizing tower system used to size the CFs (**left**), and a bar chart showing the fracture toughness of CFRPs without and with various sizing agents (**right**); reprinted from Ref. [68], Copyright 2016, Elsevier. (**b**) A schematic diagram showing the correlation between surface energy and the distribution of cross-linking points on the CFRPs (**left**), and a bar chart showing the comparative IFSS values (**right**); reprinted from Ref. [69], Copyright 2020, Elsevier. (**c**) A schematic diagram showing the CFs sizing process (**left**), and a bar chart showing the ILSS values of the various PEEK-based CFRPs (**right**); reprinted from Ref. [70], Copyright 2020, Elsevier.

Appropriate sizing can promote adhesion by supporting increased interfacial interactions that influence the mechanical properties of the CFRPs. Sizing agents can modify the CF surface and the wettability and chemical or physical reactions with polymeric matrices [64,65,66,67]. Downey et al. [68] uses aromatic and aliphatic epoxy resins as sizing agents to increase the IFSS and fracture toughness of CFRPs, as shown schematically in Figure 6a. Their results suggested that the optimization of the interface through appropriate sizing can toughen the CFRPs without significantly decreasing other static or mechanical performance. In particular, the use of an aliphatic sizing agent increased the IFSS and fracture toughness values of the CFs by ~75% and 84%, respectively, compared to the untreated CFs. Wu et al. [69] used epoxy sizing agents to enhance the surface energy and IFSS of CFRPs by regulating the number of chemical bonds at the CF/sizing agent and sizing agent/matrix interfaces. Hence, the surface energy and IFSS of the sized CFRPs were increased by up to ~20.6% and ~57.3%, respectively, compared to those of the neat CFRPs. The rate of molecular diffusion decreases as the curing and sizing agents react with the CFs and polymer matrix, such that a cross-linking density gradient is set up across the interface, with the density increasing towards the polymer matrix, as shown in Figure 6b. Moreover, the relatively small amount of DDS in the sizing agent favors the formation of a gradient interphase in cross-linking density, which is beneficial to stress transfer. The authors reported that the interfacial adhesion of the CFRPs could be optimized by adjusting the 4,4′-diaminodiphenyl sulfone (DDS) content of the sizing agent and preheating the CFs. Wang et al. [70] investigated the effects of a poly(aryl indole ketone) (PAIK) sizing agent on the ILSS of poly(ether ether ketone) (PEEK)-based CFRPs. They use a novel heterocyclic derivative of PEEK as a linkage between the CF surface and PEEK matrix through a facile and highly effective two-step method, as shown in Figure 6c. This efficient method takes advantage of the strong cation–π interactions between the PAIK and activated CFs, the increased surface roughness of the CFs, and the good compatibility between PAIK and PEEK.

These studies showed that sizing agents are effective in enhancing fiber/matrix adhesion. Nevertheless, the precise role of sizing on interface formation is unclear, particularly the degree of chemical reaction between CFs and the effects of sizing on the level of interfacial properties. Therefore, a deeper understanding of the interface between the CFs and polymer matrix is essential for achieving high-performance CFRPs.

#### 2.1.5. Coating

**Figure 7:**
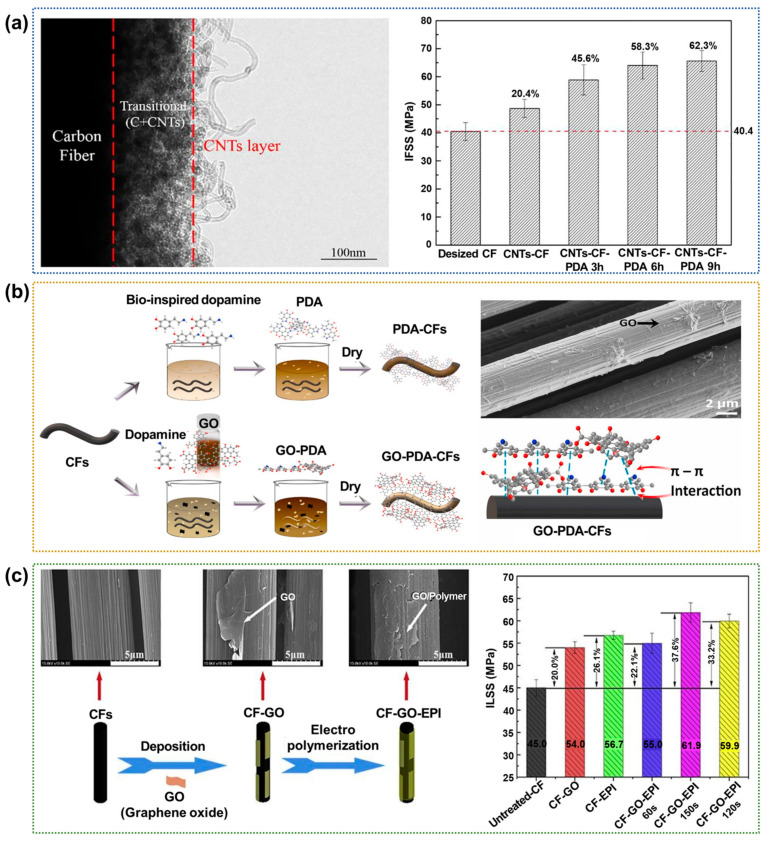
Interfacial properties of carbon nanotube-coated CFRPs: (**a**) TEM image of the CNTs grown on the CF surface (**left**) and a bar chart showing the IFSS values of various CFRPs (**right**); reprinted from Ref. [14], Copyright 2022, Elsevier. (**b**) A schematic diagram showing the surface modification of CFs with GO-PDA (**left**) and SEM image of the CFs after treatment with GO-PDA for 24 h (**right**); reprinted from Ref. [76], Copyright 2021, Elsevier. (**c**) SEM images and schematic diagrams of the CFs before and after sequential modification with GO and electropolymerization (**left**), and a bar chart showing the ILSS values of the various CFRPs (**right**); reprinted from Ref. [77], Copyright 2020, Elsevier.

Coating is the process of forming a thin, nanometer-thick layer that adheres to a substrate via physical interactions [71,72,73,74,75]. The deposition of carbon nanofillers on the CF surface is used extensively in coating technology to increase the contact area between the CFs and polymer matrix. Wu et al. [14] coated carbon nanotubes (CNTs) with polydopamine (PDA) before depositing them on the surfaces of high-strength CFs by CVD without damaging the structure of the interface or the properties of the final CFRPs (Figure 7a). The results suggested that the PDA coating facilitates a uniform distribution of the CNTs on the CF surface, effectively mitigating the corrosion of the CF in the presence of high temperature and a metal catalyst during the CVD process, enhancing the mechanical properties of the CFs and the corresponding CFRPs. The ILSS and IFSS values of the PDA/CNT-coated CFRPs increased by up to ~50.6% and ~62.3%, respectively, than the neat CFRPs. In a similar study, Zeng et al. [76] presented the self-polymerized onto the CF surface via a π–π interaction to form nano-thin surface-adherent PDA and graphene oxide (GO) layers. SEM revealed a uniform coating of GO-PDA on the CF surface (Figure 7b), suggesting that the GO-PDA treatment enhances the surface roughness of the CFs. The strong π–π interaction between the GO surface and the aromatic molecules makes the surface stacking stable against rinsing or other solution processing. Thus, the IFSS value of the CFRP was increased by 69% compared to that of the neat CFRPs after 24 h of treatment with GO-PDA. Strong π–π interactions between the GO and the aromatic molecules generally stabilize the surface stacking, enhancing the IFSS between the CFs and polymer matrix. The strong π–π interactions between the GO and the aromatic molecules generally stabilize the surface stacking, enhancing the IFSS between the CFs and polymer matrix. They reported that the polydopamine and graphene oxide surface treatments could result in the enhanced interlaminar shear strength of laminated composite structures, preventing or delaying delaminations that can happen under relatively low energy impact events. Sun et al. [77] deposited graphene oxide (GO) and the in situ electropolymerization of itaconic acid/p-aminobenzoic acid on CF surfaces to enhance the interfacial properties of CFRPs. A uniform and compact GO–polymer multiscale interfacial layer is formed on the CF surface by systematically controlling the electropolymerization time, as shown in Figure 7c. For all of the as-fabricated CFRPs, the electropolymerization time was optimal at 120s (CF-GO-EPI-120s), enhancing the ILSS by ~37.6% compared to the neat CFRPs. The authors showed that the electrophoretic deposition of GO followed by electropolymerization provides an eco-friendly and effective approach for the fabrication of CFRPs with enhanced interfacial properties.

In the above studies, neither the coating nor deposition processes modified the CFs, suggesting minimal damage to the CF surface. Thus, coating is a promising strategy for controlling the interface by grafting favorable carbon nanofillers that minimally affect the integrity of the CFs. On the other hand, a deep understanding of the interface between coating layers and polymer matrices is required to develop these strategies further.

### 2.2. Electrochemical Treatments

#### 2.2.1. Chemical Vapor Deposition (CVD)

**Figure 8:**
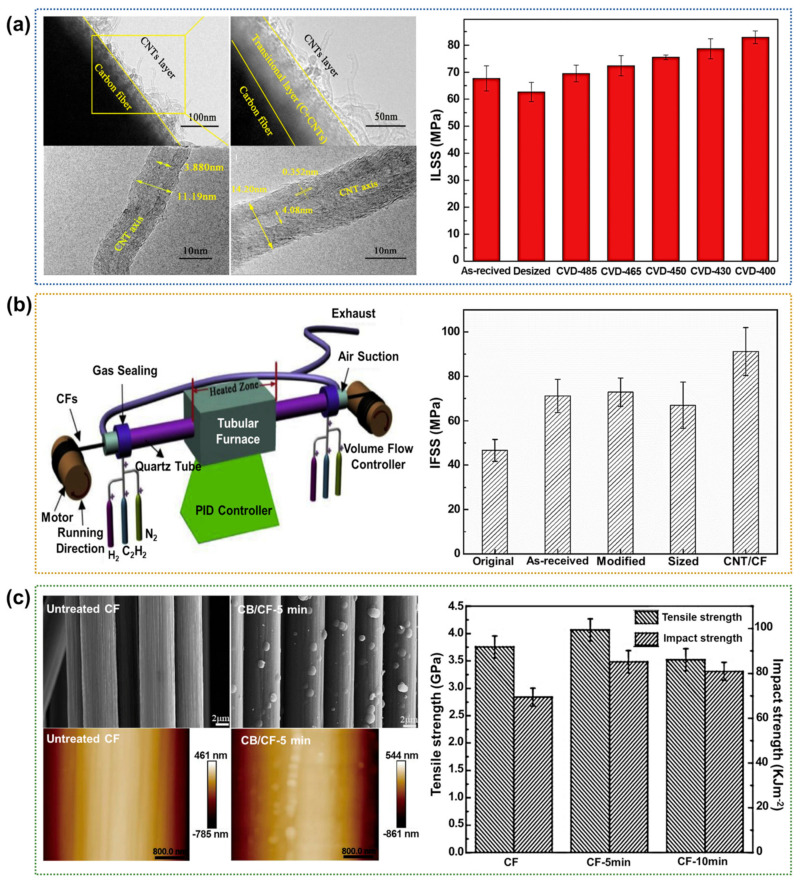
Interfacial properties of CVD-treated CFRPs: (**a**) TEM images showing the growth of CNTs on the CF surface (**left**) and a bar chart showing the ILSS values of the epoxy-based CFRPs (**right**); reprinted from Ref. [83], Copyright 2020, Elsevier. (**b**) A schematic diagram showing the equipment for the continuous in situ growth of CNTs on the moving CF surface (**left**) and a bar chart showing the IFSS values of the epoxy-based CFRPs (**right**); reprinted from Ref. [84], Copyright 2021, Elsevier. (**c**) SEM (**upper left**) and AFM (**lower left**) images showing the CFs before and after the growth of CB, and a bar chart showing the tensile and impact strengths of the epoxy-based CFRPs (**right**); reprinted from Ref. [85], Copyright 2017, Elsevier.

The CVD process is the most versatile and promising technology for direct device integration and bulk production. In this process, the CF surface is coated with a layer by a chemical reaction between the vapors of gaseous or liquid reactants [78,79,80,81,82]. Yao et al. [83] deposited CNTs on the CF surface via a novel one-step CVD method at ultra-low temperatures and used the resulting CFs to fabricate epoxy-based CFRPs. The immersion of CFs in a catalyst solution with a 1:1 molar ratio of Fe(NO_3_)_3_·9H_2_O and Co(NO_3_)_2_·6H_2_O, followed by processing in a CVD furnace, represents a critical step in the fabrication process. During the growth of CNTs, C_2_H_2_ and H_2_ were introduced at temperatures of 485, 465, 450, 430, and 400 °C, with the gas flow ratio of N_2_, C_2_H_2_, and H_2_ controlled at 10:5:5 L/min, while the furnace pressure was consistently maintained at 0.01 MPa. Various temperatures of 485, 465, 450, 430, and 400 °C were used to obtain the CVD-treated CF samples designated as CVD-485, CVD-465, CVD-450, CVD-430, and CVD-400, respectively. The CNTs grew from the CF surface (Figure 8a), and were embedded in amorphous carbon to form a transitional layer that anchored them firmly to the CF surface. The results suggested that the optimal CVD temperature was 400 °C, which led to ~32.3% enhancement in the ILSS relative to that of the neat CFRPs. In another study, Qin et al. [84] reported the in situ growth and uniform distribution of CNTs on the surface of continuous CFs. The chemical reduction of catalyst particles and the growth of CNTs are performed under ambient pressure using a unique open CVD furnace, as illustrated in Figure 8b. The reduction of catalysts (Co(NO_3_)_2_) and the growth of CNTs were conducted under ambient pressure in a CVD furnace, with nitrogen (N_2_) purging to maintain an inert gas atmosphere throughout the process. The catalyst precursors were converted into particles at 450 °C using a mixed gas of H_2_ and N_2_, followed by the introduction of C_2_H_2_, H_2_, and N_2_ into the CNT growth zone at 600 °C, where CNTs were grown in situ on the CF surface, with flow rate ratios of 0.3:0.6 L min^−1^ for the reducing gas and 0.15:0.15:0.3 L min^−1^ for the CNT growth gas. For the fiber specifications used in the experiment, the output was approximately 7.2 g/h, and the entire experiment process lasted for 5 h. This enhanced the mechanical properties, with a ~95.4% increase in the IFSS compared to that of the neat CFRPs. They reported that the uniform distribution of CNTs at the interface of the epoxy-based CFRP provided effective stress transfer. Dong et al. [85] enhanced the mechanical properties of epoxy-based CFRPs without sacrificing the tensile strength of the base fibers using thermal CVD to grow carbon black (CB) on the CF surface (Figure 8c). In their study, the graphite frame was positioned in a single-zone atmospheric pressure quartz tube furnace with a 60 cm long heating zone, where a constant nitrogen flow of 1000 sccm was introduced into the quartz tube for 10 min in order to exhaust the air. Then, the reactor temperature was increased to 1000 °C at a rate of 10 °C/min, and ethanol was introduced into the reactor at a flow rate of 20 mL/h via a pump to facilitate the growth of CB on the CFs. The temperature of the reactor was increased to 1000 °C at a heating rate of 10 °C/min, and N_2_ was used as the carrier gas at a flow rate of 400 sccm during the deposition process. The untreated and treated CFs were denoted as CF and CF-X min, where X corresponds to the CB growth time. The tensile and impact strength values of the CF-5 min increased by ~4.3% and 16.6%, respectively, than the CFs. The authors concluded that the CB on the CF surface functioned as an interface shielding layer that relieved the stress concentration and prevented the crack tips from deviating from the crack pathway.

The CVD process endows the deposited layer with a directional and controllable size distribution and orientation on the CF surface, enhancing the mechanical properties of the interface between the CF and polymer matrix. Nevertheless, the higher growth temperature required by CVD tends to cause oxidation and damage to the CF surface, leading to a decrease in bulk strength. Hence, the fabrication of an orderly, controllable, and stable layer on the CF surface while effectively avoiding damage to the fiber strength is essential for realizing the advantages of toughened CFRPs.

#### 2.2.2. Electrophoretic Deposition (EPD)

**Figure 9:**
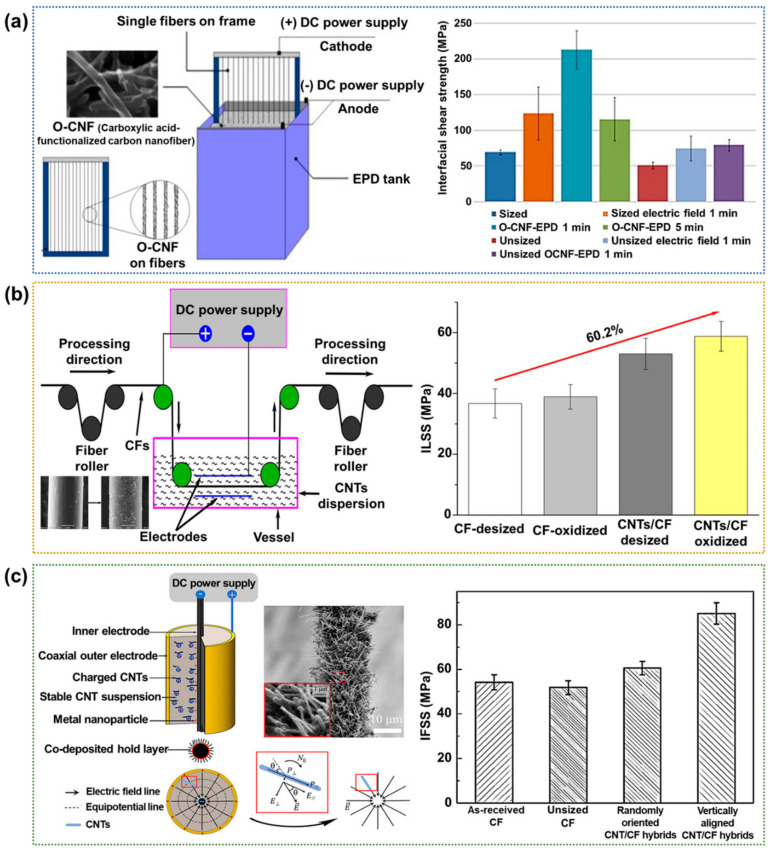
Interfacial properties of EPD-treated CFRPs: (**a**) SEM image and schematic diagram of the frame-mounted CFs being lowered into the EPD tank containing an O-CNF solution (**left**) and a bar chart showing the ILSS values of the various epoxy-based CFRPs (**right**); reprinted from Ref. [91], Copyright 2011, Elsevier. (**b**) A schematic diagram of the continuous EPD deposition of CNTs onto the CF surface (**left**) and a bar chart showing the ILSS values of the epoxy-based CFRPs (**right**); reprinted from Ref. [92], Copyright 2015, Elsevier. (**c**) A schematic diagram showing the alignment of CNTs onto the CFs (**left**) and a bar chart showing the comparative IFSS values (**right**); reprinted from Ref. [93], Copyright 2020, Elsevier.

The EPD process is an important technique for depositing CFRPs from colloidal suspensions or solutions of macromolecules. The EPD process allows the outstanding control of the layer thickness and deposition rate [86,87,88,89,90]. Schaefer et al. [91] deposit carboxylic acid-functionalized carbon nanofibers (O-CNFs) on the CF surface to enhance the interfacial properties of the resulting CFRPs, as shown schematically in Figure 9a. Here, the EPD was conducted by dispersing 6.5 mg of O-CNFs in 300 mL of ultrapure water using sonication (15 min at 140 W), followed by the addition of 800 mL of ultrapure water to create a final O-CNF concentration of 5.9 μg/mL in an electrophoretic tank. After 1 and 5 min of O-CNF deposition, the CFs exhibited 207.6% and 66.9% increases in the IFSS value, respectively, compared to that of the untreated CFs. The authors attributed this increase to enhancements in the surface roughness and surface area because of the uniform deposition of closely bound short and long O-CNFs in axial alignment with the CFs. Deng et al. [92] deposit CNTs onto the CF surface via oxidative treatment combined with electrophoretic deposition, as shown schematically in Figure 9b. For the EPD of CNTs onto CFs, the CF tow served as the deposition electrode, while two 50 × 20 cm^2^ stainless steel plates were placed opposite the CFs as the counter electrodes, with a CNT concentration of 0.05 g/L. The EPD process was conducted at a constant voltage of 20 V for 20 min, with an electrode distance of 2 cm and a CF tow length of 50 cm, which was wound up on a roll. The deposition of CNTs introduced some polar groups on the CF surface, enhanced the surface roughness, and altered the surface morphology of the CFs. The ILSS value of the CNT-deposited CFRP was increased by 60.2% compared to that of the neat CFRPs because of the introduction of this new interface layer. Li et al. [93] prepared vertically aligned carbon nanotube/CF (VACNT/CF) hybrids by EPD under mild conditions. A coaxial cylindrical anode and a CF filament cathode were used to ensure the deposition of dense VACNT arrays on the CF surface at low deposition voltage (Figure 9c). CNTs were dispersed in an organic solvent by sonication for 30 min, with the addition of Mg(NO_3_)_2_·6H_2_O to positively charge the CNTs for the cathodic process. Thus, CNTs were positioned at the adsorbed locations with the metal nanoparticles as a co-deposited holding layer. The contact angle and IFSS of the vertically aligned CFs were enhanced by 48.3% and 58.1%, respectively, compared to that of the neat CFRPs. The authors reported that the polymer matrix infiltrated the VACNT arrays through capillary action, which were mechanically interlocked with the polymer matrix.

Compared with other surface treatments, EPD is a simple process with a high deposition rate, environmental friendliness, good surface uniformity, and controllability by varying the process parameters (e.g., suspension concentration, time, and voltage). On the other hand, using the EPD technique, the physical adsorption between the CF and coating was weak, so the coating was easily detached from the CF surface. Hence, multi-dimensional layers must be introduced simultaneously onto the fiber surface, and a CFRP interface must be designed to utilize the advantages of the EPD process.

### 2.3. Dry Treatments

#### 2.3.1. Plasma Treatment

**Figure 10:**
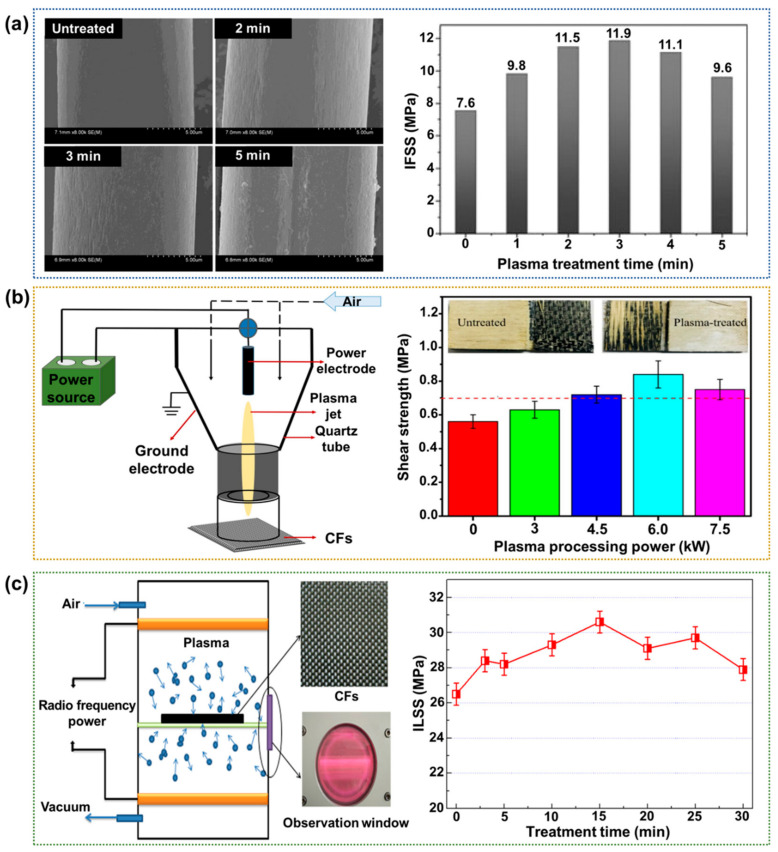
Interfacial properties of plasma-treated CFRPs: (**a**) SEM images of the CF surfaces before and after various treatment times (**left**) and a bar chart showing the IFSS values of the bismaleimide-based CFRPs after various treatment times (**right**); reprinted from Ref. [97], Copyright 2018, Wiley. (**b**) A schematic diagram of the APPJ system (**left**) and a bar chart showing the shear strengths of the formaldehyde-based CFRPs after various treatment times (**right**); reprinted from Ref. [98], Copyright 2019, Elsevier. (**c**) A schematic diagram of CFs in a plasma processor (**left**) and a plot of the ILSS value against treatment time for the vinyl ester-based CFRPs (**right**); reprinted from Ref. [99], Copyright 2020, IOPscience.

Plasma technology is a well-known dry treatment favored for its operational simplicity, short modification time, and eco-friendly processes. In particular, the method does not produce any waste or use harmful chemicals. In addition, this method can make the surface more hydrophilic by introducing oxygen-containing functional groups and increasing the surface roughness [94,95,96]. Zhuoda et al. [97] reported continuous CF surface modification via radio frequency (RF) inductively coupled plasma (ICP) and dielectric barrier discharge (DBD) low-temperature plasma treatments. After the plasma treatment, the surface roughness of the fiber increases sharply, enhancing the wetting properties, as shown in Figure 10a. For all the fabricated CFRPs, a low pressure, low power, and moderate treatment time (3 min) have the optimal effect on the modification of the CFRP interface, enhancing the IFSS value by ~57.0% compared to that of the neat CFRPs. Zhang et al. [98] used an atmospheric pressure plasma jet (APPJ) treatment to enhance the interfacial adhesion between the CF and adhesive for the preparation of veneer-based CFRPs with outstanding mechanical performance. As a result, the distilled water contact angle of the CF decreased by 49.7%, and the surface energy increased by 41.3% after plasma modification at an optimal processing power of 6 kW. Moreover, the shear strength was enhanced by ~50.0% compared to that of the neat CFRPs (Figure 10b). They reported that the APPJ plasma could become a promising technology for improving the interfacial adhesion of CF and adhesiveness to manufacture the CFRPs. Kong et al. [99] examined the use of low-temperature plasma treatment for enhancing the ILSS of vinyl ester resin (MFE-5)-based CFRPs. This treatment enhances the CF wettability significantly at an airflow rate of 150 sccm and a discharge power of 200 W, as shown in Figure 10c. The ILSS value of CFRP was ~15.4% higher after a treatment time of 15 min than neat CFRPs.

The above examples show that plasma technology is promising for modifying CFs, leading to enhanced interfacial properties in the corresponding CFRPs. On the other hand, although the exposure time and plasma intensity are often highly controlled, excessive oxidation can compromise the inherent strength of the CFs.

#### 2.3.2. Ozone Treatment

**Figure 11:**
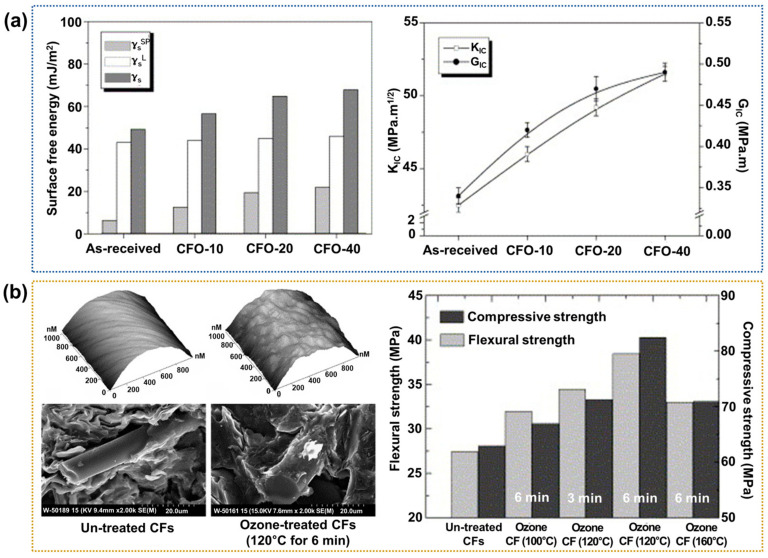
Interfacial properties of ozone-treated CFRPs: (**a**) Variations in the surface free energy (**left**) and fracture toughness (K_IC_) of epoxy-based CFRPs according to the ozone treatment time (**right**); reprinted from Ref. [103], Copyright 2005, Elsevier. (**b**) AFM and fracture surface (**left**), and a bar chart showing the flexural and compressive strengths of CFRPs under various treatment conditions (**right**); reprinted from Ref. [104], Copyright 2006, Elsevier.

Ozone treatment is regarded as an excellent method because it can easily provide oxygen radicals in an O_3_ atmosphere without incurring defects [100,101,102]. For example, Park et al. [103] investigated the effect of ozone treatment at concentrations of 10, 20, and 40 mgL^−1^ on the surface energy and fracture toughness (K_IC_) of the resulting epoxy-based CFRPs (designated as CFO-10, CFO-20, and CFO-40, respectively). The experiments were performed at a constant temperature of 25 °C, an operating voltage of 220 V, and a pressure of 60 psi, with oxygen as the purge gas. The results indicated that both the surface energy and K_IC_ were increased with increasing ozone concentration, with increases of ~55.0% and ~25.0%, respectively, for the CFO-40 compared to the neat CFRP (Figure 11a). The authors concluded that the increased number of oxygen-containing functional groups generated by the ozone treatment play an important role in the increased interfacial adhesion between the CFs and polymer matrix, thereby enhancing the mechanical performance of the CFRPs. Jin et al. [104] reported that the ozone treatment of CFs enhances the compressive strength and flexural strength of CFRPs. In this case, the optimal treatment conditions were 6 min at 120 °C, showing ~46.1% and 31.7% increases in the flexural and compressive strengths, respectively, compared to the neat CFRPs (Figure 11b). These increases were attributed to the increased number of oxygen-containing functional groups and surface roughness of the CFs due to the ozone oxidation method.

Accordingly, ozone-treated CFRPs with superior interfacial interaction are promising candidates for developing new advanced composites. Nevertheless, technical difficulties, such as processing capability, limit their large-scale applicability.

### 2.4. Polymer Matrix Modifications

#### Addition of Nanofillers to the Polymer Matrices

**Figure 12:**
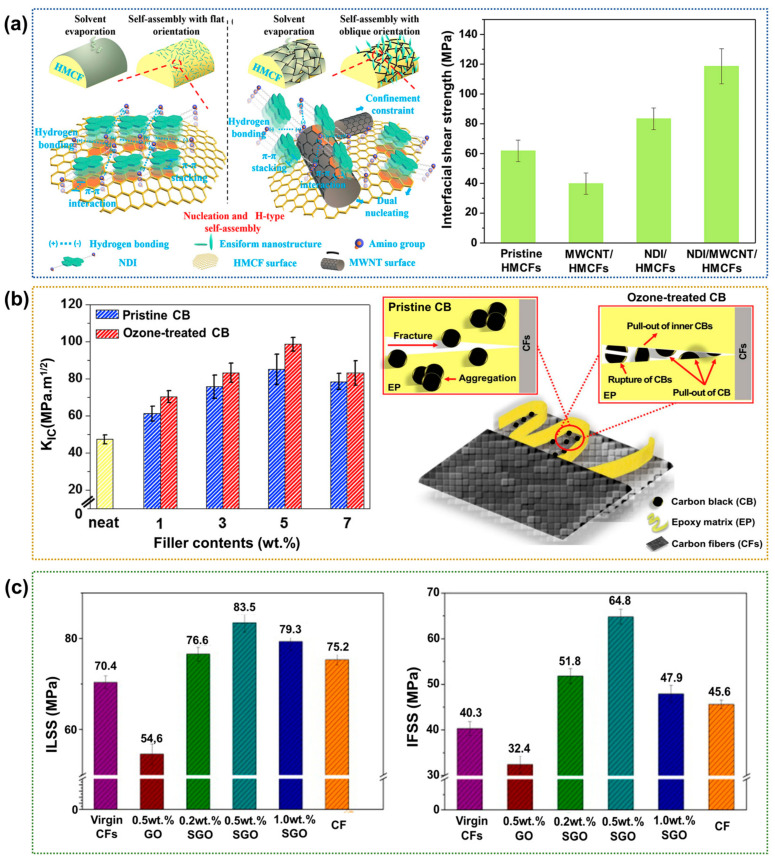
Interfacial properties of nanofiller-based CFRPs: (**a**) Schematic diagrams showing the self-assembly of NDI/MWCNT/HMCF (**left**) and a bar chart showing the IFSS values of the various CFRPs (**right**); reprinted from Ref. [108], Copyright 2019, Elsevier. (**b**) A chart showing the fracture toughness (K_IC_) values (**left**) and schematic diagrams showing the cracking mechanism of the OCB loaded CFRPs (**right**); reprinted from Ref. [109], Copyright 2019, Elsevier. (**c**) Bar charts showing the ILSS values (**left**) and IFSS values (**right**) of CFRPs with various concentrations of SGO; reprinted from Ref. [110], Copyright 2014, Elsevier.

One of the most effective polymer matrix modification methods involves fabricating a multiscale structure with synergistically integrated nanoscale fillers, such as CNTs, CB, and graphene, along with macro-scale fibers in the CFRPs [105,106,107]. Lin et al. [108] examined the interfacial properties and reinforcing mechanisms of a multiscale interface consisting of a high-modulus carbon fiber (HMCF) supporting self-assembled amino terminated, π-conjugated naphthalenediimide (NDI) on multi-wall carbon nanotubes (MWCNTs). As shown in Figure 12a, in the absence of MWCNTs, the NDI nanostructures are deposited horizontally on the HMCF surface. By contrast, oblique ensiform NDI nanostructures are also deposited on MWCTs when MWCNTs are present on the HMCF surface, leading to the construction of multiscale NDI/MWCNT/HMCF interfaces. As a result, the IFSS of the NDI/MWCNT/HMCF/epoxy composite is ~91.8% higher than that of the neat CFRPs, confirming the positive effect of the self-assembled nanostructure on the interfacial adhesion. Kim et al. [109] examined the reinforcement influence of varying amounts of ozone-treated CB (OCB) in the CFRP interlayer. The optimal OCB content was 5.0 wt.%, which increased the fracture toughness by ~121.5% compared to that of the neat CFRPs. In addition, interfacial interactions in the CFRPs were enhanced significantly after the ozone treatment for 4 h because of the introduction of carboxyl groups. Figure 12b presents a schematic diagram of the proposed mechanism behind the fracturing and post-crack toughness of the CFRPs. The authors suggested that the untreated (pristine) CB particles aggregate in the epoxy matrix, while the OCB particles bridge the crack but can experience pull-out or even a complete rupture. Chen et al. [110] examined the reinforcement influence of varying amounts of silanized GO (SGO) in the CFRP interlayer. The optimal silanized GO content is 0.5 wt.%, leading to ~18.6% and 60.8% higher ILSS and IFSS, respectively, than the neat CFRP, as shown in Figure 12c. They concluded that the chemical interactions between the amino groups of the silane coupling agent and the epoxy matrix enhanced chemical adhesion between the SGO and epoxy matrix.

**Figure 13:**
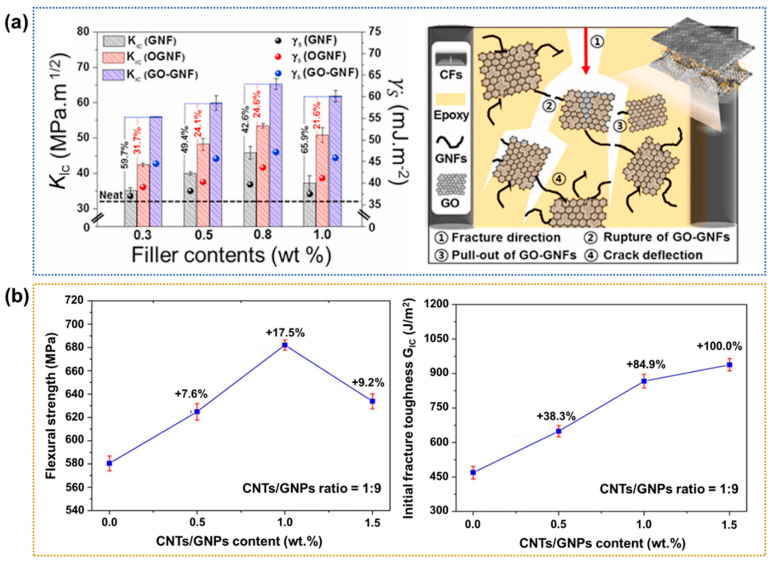
Interfacial properties of nanohybrid-based CFRPs: (**a**) Var chart showing the K_IC_ values at various filler contents (**left**) and a schematic diagram of the proposed fracture mechanism in the GO-GNF-loaded CFRP (**right**); reprinted from Ref. [115], Copyright 2021, Elsevier. (**b**) Plots of the flexural strength (**left**) and initial fracture toughness (**right**) of the CFRPs with various loadings of CNTs/GNPs; reprinted from Ref. [116], Copyright 2015, Wiley.

Hybridizing carbon nanofillers to produce a multiscale hierarchy is an innovative and effective approach for significantly enhancing the interfacial properties of CFRPs by enhancing the interfacial interlocking between the CFs and polymer matrix [111,112,113,114]. Kim et al. [115] proposed an efficient method for the deep and stable penetration of graphene oxide/graphitic nanofiber nanohybrids (GO-GNFs) into the epoxy matrix of CFRPs. The ILSS and K_IC_ values of the 0.8 wt.% GO-GNF-loaded CFRPs were ~159.5% and 102.8% higher, respectively, than those of the neat CFRPs. Figure 13a presents the mechanism of the improved mechanical properties via interfacial interlocking. They reported that the crack propagation through the epoxy matrix occurs because cracks must pass through or around the GO-GNF-rich interlayers, resulting in enhanced ILSS and K_IC_ values. In a similar study, Wang et al. [116] examined the reinforcement effect of various amounts of CNT/graphene nanoplatelet (CNTs/GNPs) nanohybrids in a CFRP interlayer. The results indicate an optimal GO content of 1.0 wt.%, which enhances the flexural strength and initial fracture toughness by ~17.5% and 84.9%, respectively, compared to those of the neat CFRPs, as shown in Figure 13b. They concluded that the CNTs/GNPs hybrids can increase the interfacial friction between the CFs and matrix, enhancing the mechanical properties of the CFRPs.

In summary, the deformation of the polymer matrix by the addition of nanofillers enhances the interfacial interaction between the CFs and polymer matrix, promoting crack propagation in the fiber–matrix structure. However, a significant challenge in using nanofillers in CFRPs is their high hydrophobicity and low dispersibility, which can result in irreversible agglomeration over time due to van der Waals interactions between the prismatic surfaces. Thus, it is essential to recognize that enhanced mechanical properties can be achieved by increasing the nanofiller content within a reasonable range. The concurrent modification of nanofillers and polymer matrix, rather than just the fiber–matrix interface itself, presents a promising area of research for understanding the generation of interfaces.

## 3. Perspectives and Conclusions

Carbon fibers (CFs) are promising reinforcing materials in polymer-based composites that are valued for their exceptional thermal stability, chemical resistance, and mechanical properties. This review explored the recent research progress on the application of CFs as reinforcement materials in polymer-based composites, with a focus on the interfacial behavior of carbon fiber-reinforced polymer composites (CFRPs). In this regard, various surface treatment techniques, including wet, electrochemical, and dry processes, as well as methods for modifying the polymer matrix by adding nanofillers, were reviewed (Table 1). Although wet and electrochemical surface modification techniques are effective, they pose environmental concerns because of the use of harmful chemicals, and their high cost and low yields limit their practical applicability. Dry surface modification techniques require specialized equipment and complex conditions, such as specific power levels, gas types, and treatment durations, which hinder their large-scale applicability. Therefore, the modification of the polymer matrix by the incorporation of nanoscale fillers is a promising strategy for improving the interfacial properties of the CFRPs. The addition of nanofillers produces unique hierarchical structures that improve the compatibility of the CFRP components by increasing the interfacial interactions between the matrix and carbon fibers. On the other hand, the addition of nanoscale fillers may increase the cost and is currently constrained by technological limitations in controlling the CFRP process. Thus, innovative advanced processing and functionalization modification technologies are crucial for enhancing the comprehensive properties of CFRPs to meet the complex demands of emerging and diverse industries. Based on the current research status of surface modifications of CFRPs, the following relevant conclusions and perspectives are drawn: (1) Combining multiple surface modification techniques is essential to prevent the loss of mechanical properties. (2) Optimizing methods for the interface to achieve outstanding mechanical properties, while addressing issues such as toxicity, expensive reagents, process cost reductions, and the use of fewer materials, should be developed to accelerate the process. (3) Various materials, such as macro-, micro- or nano-scale fillers should be introduced at the same time to construct multiscale interface layers to increase the interfacial layer thickness and balance the interfacial interaction between CF and polymer matrix and to increase the effective load transfer for reduced stress concentration. Overall, the above discussion on the various strategies for improving the interfacial properties is expected to guide future research directions for advanced CFRPs, including providing a theoretical understanding of the interfacial composition and structural design process, which could potentially lead to significant breakthroughs in the field of CFRPs.

## Figures and Tables

**Table 1:** A summary of CFRP modification strategies.

Methods	Processing Method	Improvement	Features	Limitations	Ref.
Acid	HNO_3_/H_2_SO_4_ (3:1)	IFSS 37.8 MPa (29.2%)	Relatively high efficiency in surface roughness	Long-term acid treatment may cause defects or reduce the strength Cost for refining	[51]
	HNO_3_	ILSS 58.2 MPa (74.6%)			[52]
Amine	Poly(oxypropylene) diamine	IFSS 83.8 MPa (79.1%)	Good interface compatibility	Long reaction time Harmful acid–base solutions	[57]
	Poly (amido amine) (PAMAM) dendrimer	ILSS 81.5 MPa (53.1%)			[58]
Silane	3-aminopropyltriethoxy silane (KH550)	IFSS 70.5 MPa (73.1%) ILSS 81.3 MPa (61.2%)	Silane coupling agents bond organic materials to inorganic materials	Difficult to dry Need for high concentration of silane coupling agents	[62]
	[3-(2-Aminoethyl) aminopropyl] trimethoxysilane	IFSS 30.0 MPa (47.9%)			[63]
Sizing	Aliphatic epoxy (polypropylene glycol diglycidyl ether)	ILSS 53.6 MPa (115.2%)	Not cause damage to the fiber	Complex and difficult for optimization process	[68]
	Diglycidyl ether of Bisphenol A (E51)	IFSS 122.4 MPa (57.3%)			[69]
	Poly(aryl indole ketone)	ILSS 46.9 MPa (61.7%)			[70]
Coating	CNT	ILSS 89.9 MPa (50.6%) IFSS 65.7 MPa (62.3%)	Not cause damage to the fiber	Requires a deep understanding of the interface/interphase of multiple structures	[14]
	GO	IFSS 14.7 MPa (69.0%)			[76]
CVD	CNT	ILSS 82.9 MPa (32.3%) IFSS 67.5 MPa (30.7%)	Orderly and controllable Not cause damage to the fiber	High cost Dependent on various parameters	[83]
	CNT	IFSS 91.2 MPa (95.4%)			[84]
Plasma	Dielectric barrier discharge (DBD) low-temperature plasma	IFSS 11.9 MPa (57.0%)	Simple and eco-friendly processes	Dependent on various parameters	[97]
	Atmospheric pressure plasma jet	ILSS 0.8 MPa (50.0%)			[98]
Addition of nanofiller	CNT	IFSS 118.6 MPa (91.8%)	Large surface-to-volume ratio of particle–matrix interactions	Difficult to disperse in polymer matrix	[108]
	Carbon black (CB)	ILSS 35.7 MPa (70.0%)			[109]
	GO-GNF hybrid	ILSS 43.5 MPa (159.5%)			[115]

## Data Availability

Not applicable.
